# Keyword Index for Volume 107

**DOI:** 10.1038/bjc.2012.533

**Published:** 2012-12-04

15-PGDH 707, 1514

25-hydroxyvitamin D 1589

2D : 4D 1631

5-ALA 462

5-aza-2′-deoxycytidine 1116

5-fluorouracil 435

5FU toxicity 1525

6th vital sign 617

*α*-carotene 1580

accelerated-failure time 531

active surveillance 1467

activin 1978

activin receptor 1978

acute pain 937

ADAMs 143

adenocarcinoma 143

adenocarcinoma *in situ* 1451

adenovirus 448

adherence to therapy 904, 1249

adhesion 129

adjuvant chemotherapy 1257, 1678

adult height 165

advanced NSCLC 442

advanced urothelial carcinoma 1031

adverse event 612

aflibercept 598, 604

Africa 1624

age at first birth 564

AhR 43

AIS 1451

Akt 847

Akt1 713

albumin 275

alcohol consumption 234, 537, 879

alternative transcript use 1409

ambulatory patients 1244

amino-acid transporter 632

AML 108

amplification 1997

anal carcinoma 1864

anastrozole 1815

angiogenesis 598, 604, 700, 977, 1207, 1227, 1481, 1498,

angiogenesis inhibitors 455

angiogenic factors 501

animal models 1944

anthracyclines 1257

anticancer drugs 1100, 1797

anti-EGFR antibody 340

antitumour activity 53

anti-vascular therapy 469

anti-VEGF antibody 308

anti-VEGF therapy 598

anxiety 1459, 1644

apolipoproteins 1883

apoptosis 63, 469, 1518

artemis overexpression 1506

Asian 1075

aspirin 1514, 1602

ATR 291

atrophic gastritis 888

autopsy 1

axitinib 1268, 1277

AZD8055 1093

BARD1 isoforms 675

basal core promoter mutant 2010

basement membrane 1153

BCAR4 947

*β*-carotene 1580

*β*-catenin 1144, 1514

Bcl-2 1714

*BCL2* 967

benefits and risks 1938

benign tumours 1761

bereavement 544

beta1 integrin 1969

bevacizumab 287, 360, 658, 961, 1481

BI 2536 280

bicalutamide 1714

*β*III-/*β*V-tubulin 823

bilateral breast cancer 549

biomarker 71, 508, 823, 840, 1009, 1031, 1354, 1384, 1518, 1729, 1754, 1883, 1987

biomarker mRNA 150

bisphosphonates 7, 1665

bladder cancer 116, 123, 469, 477, 1384, 1392, 1826

body mass index 169, 1608

bone 63, 1547

bone marrow endothelial cells 1737

bone marrow stroma 1737

bone matrix 713

bone metastases 646, 1665

bortezomib 1844

brain metastases 1454, 1075

brain-only metastatic behaviour 1454

*BRCA1* 675, 2005

*BRCA1/2* mutation 24

*BRCA2* 2005

breast biomarkers 382

breast cancer 7, 12, 18, 24, 63, 71, 75, 129, 230, 375, 527, 724, 732, 748, 793, 800, 874, 947, 956, 1175, 1239, 1257, 1459, 1584, 1608, 1631, 1722, 1815, 1892, 1901, 2016

breast cancer risk 176

breast cancer subtypes 1454

breast cancer surgery 937

breast masses 224

breast surgery 1459

Breslow thickness 570

C75 300

caffeine 874

calcium channels 375

calibration 1025

calprotectin 1876

CALYPSO 588

cancer cells 1337

cancer clinical trials 1017

cancer prevalence 1195

cancer progenitors 43

cancer registration 1159

cancer screening 1938

cancer therapy 1207

cancer vaccine 1302

capecitabine 585, 1268, 1678

carbonic anhydrase IX 1131

carboplatin 442, 588, 1310

carcinoembryonic antigen-related cell adhesion molecule 1745

carcinoma 739

carotenoids 1580

case–control study 1589, 1644

castration-resistant 592

CD31 1044

CD98 632

CDK 516

CEA 266, 724

CEBPD 732

CECs 1564

cell cycle 1083

cell death 469

cell fraction 1345

cell lineage 2016

cell-cycle arrest 516

cellular radiosensitivity 1506

CEPs 1564

cervical cancer 243, 556, 918, 1445, 1574, 1917, 1956

cervical neoplasia 1624

cervical screening 243

cervix 739

cetuximab 626, 1932

chemokine 1729

chemokines/receptors 1944

chemotaxis 713

chemotherapy 7, 31, 158, 531, 549, 840, 1100, 1107, 1277, 1327, 1637, 1826

Chernobyl 994

chick chorioallantoic membrane assay 1853

childhood 183

childhood cancer 544, 1159, 1652

childhood cancer survivors 234

childhood leukaemia 1159, 1163, 1652

childhood obesity 169

choriocarcinoma 1969

chromosome instability 71

chromosome translocation 739

chronic myeloid leukaemia 904

CIN 1451

circulating endothelial cells (CEC) 1286

circulating tumour cells (CTC) 448, 1286

circumferential resection margin 1925

cirrhosis 195, 2010

cisplatin 31, 360, 604, 967, 1853

CK19 724

classical CMF 1257

cleaved tRNA 1987

clinical trials 808, 910, 1017, 1672

clonality 2016

cluster analysis 234

coffee 874

cohort 195, 1181

collagen type IV 1153

colon cancer 352, 1169, 1678, 1944

colonoscopy 417, 765

colonoscopy/^*^methods 1051

colorectal cancer 189, 260, 287, 340, 345, 417, 626, 667, 675, 757, 765, 831, 931, 1001, 1037, 1044, 1518, 1525, 1584, 1602, 1729

colorectal cancer mortality 255

colorectal cancer prevention 1514

colorectal cancer screening 1295

colorectal neoplasm 150, 417, 1051

combination antiretroviral therapy 1624

combination treatment 1692

co-morbidity 189, 1175, 1901

conditional power 910

CONFIRM 1044

connexin-26 1963

continual reassessment method 1022

contralateral breast cancer 221

contralateral testicular germ cell tumour 1637

copper 1488

costs 1574

COX-2 inhibitor 707

C-reactive protein 275, 1031

creatine kinase 1797

CRP 1131

cryotherapy 772

CT genes 116

cumulative incidence 1637

curcumin 1083

CXCR4 43, 491

CXCR7 antagonists 1944

CYC3 1692

cyclin D1a 1684

cyclin D1b 1684

cyclins 516

cyclodextrin 1083

cyclooxgenase 207

CYP2D6 polymorphism 230

cytokine 639, 1729

cytology 1384

daytime napping 527

DCIS 1451

debulking surgery 1069

decision curve analysis 1031

decision tree analysis 1025

deep vein thrombosis 1244

defective DNA repair 1506

degradation 684

degradome 143

delayed diagnosis 1159

demographics 1810

dental health 888

depression 1644

deprivation 765

developing countries 1445

diabetes 1608

diagnosis 260, 1220, 1876

diagnostics 1987

diet 537

dietary cadmium 895

differential splicing 675

digit ratio 1631

direct sequencing 626

discrimination 1025

disease outcome 370

disseminated tumour cell 1345

disulfiram 1488

DJ-1 1595

DLBCL 491

D-loop 2016

DNA double-strand break repair 1776

DNA hypomethylation 1116

DNA microarray 1433

Doc 814

docetaxel 435, 1233, 1268, 1474

dose banding 1100

dose escalation 1022

dose standardisation 1100

dose–response relationship 1188

dosing regimen 455

doxorubicin 37

DPD 1950

drug resistance 1399

dysregulated pathways 1722

early breast cancer 1249

EDTA 1310

education 189

*EGFR* 1864

EGFR inhibitor 1745

elastography 224

elderly 570

emergency 1220

EMMPRIN 667

EMT 707

endocrine therapy 549, 1249

endometrial cancer 84, 169, 370, 1997

endothelial cell 1207

EORTC 1

Ephrin A2 1737

epidemiology 176, 183, 195, 874, 895, 1188, 1451, 1652, 1901

epidermal growth factor receptor (EGFR) 1745

epigenetics 75, 375, 1423

epirubicin 71, 1257

epithelial ovarian cancer 1783

epothilones 808

ERBB2 947

ERCC1 1950

erlotinib 1820

erythropoietin 1317

erythropoietin receptor 1317

ESCC 1233

estrogen receptor signalling 1722

ethics 1022

ethnic minorities 1017

ethnicity 1908

*EVX1* 100

exact tests 1801

exon arrays 1409

faecal immunochemical test 1295

faecal occult blood testing 255

FAK 856

fatty acid 129

fatty acid synthase 300, 977

female breast cancer 165

fetal haemoglobin 477

fetal haemopoiesis 477

FFA2/FFAR2/GPR43 1337

FFPE 684

fibroblast growth factors 1327

fine-needle aspirates 1354

fixed dose 1100

flexible sigmoidoscopy 1295

fluorescent *in situ* hybridisation (FISH) 71

FOBT 1938

FOLFOXIRI 1932

FOXM1 1766

futility analysis 910

G2–M cell-cycle phase 1766

gambogic acid 1702

gastric adenocarcinoma 1125

gastric cancer 31, 137, 143, 275, 1584, 1595, 1908

GBM stem-like cells 1488

gemcitabine 411, 442, 1083, 1488

gene amplification 325

gene expression 994, 1433

gene expression microarray 508

gene expression profiling 91, 1116

genetic alterations 1418

genetics 748

genomic biomarker 340

genomic profile 1418

geographical differences 1595

germ cell tumour 1833

gestational trophoblastic tumours 1810

GFP 448

GIST 1433

Gleevec 592

glioblastoma 1481

glomerular filtration rate 1310

GPRD 1602

granulocyte colony-stimulating factor 814

*GRIA2* 91

gut microbiota 1337

gynaecological cancers 448

hand–foot syndrome 585, 1678

HbF 477

HBV DNA levels 2010

HBV genotypes 2010

head and neck cancer 1138

health behaviour 201, 234

health inequalities 183

hematopoietic stem cells 1302

hemodynamic response imaging 658

heparins 1244

hepatocellular carcinoma 988, 1672, 2010

HER2 800, 956, 1302

HER2-positive 793

HER2-positive breast cancer 1075

hereditary non-polyposis colorectal cancer 1783

heterogeneity 1392

*HGF* 793

high-resolution melting analysis 1864

histology 165

histone methylation 315

HIV 1624

HIV/AIDS 556

HNSCC 707

Hodgkin's disease 531

homologous recombination deficiency 1776

hookah 1618

hormone naïve prostate cancer 1554

hormone receptor 7

hormone refractory 592

hormone status 864

hormones 1631

hospital admissions 1213

host cells 1345

hot flushes 230

HPV 1917

HPV infection 482

HPV vaccination 1445

HRMA 626

Hsp27 63

huachansu 411

human chorionic gonadotropin 1969

human glioblastoma 462

human immune system mice 1302

human papillomavirus 243, 556, 1574, 1624

humans 1051

hypercapnia 658

hyperoxia 658

hypertension in pregnancy 176

hypoxia 291, 516, 1714

IHC 1684

IL-12 1302

IL-12p40 1956

IL-6 1956

imaging 224

imatinib 592, 1433

immune suppression 1869

immunocytochemistry 1384

immunoexpression 370

immunohistochemistry 84, 325, 1745, 1864

immunoproteasome 53

immunotherapy 116, 1116

incidence 575, 1159, 1644, 1908, 2005,

Indonesia 772

infection 931, 1163

inflammation 1534

inflammation-based prognostic score 988

inhibin B 1833

inhibitor 53

INRG 1418

integrin *α*_v_*β*_3_ 713

integrins 847

interviews as topic/methods 1051

inulin-type fructans 1337

invasion 129, 713, 739, 1374, 1969

irinotecan 435

ITGA9 1374

*K- ras* 1864

KDR 1044

Ki-67 632

*KRAS* 340, 1997

KRAS 626

*KRAS* mutation testing 345

lactate dehydrogenase 422

laminin 1153

lapatinib 956, 1075

LAT1 632

late effects 1833

leg length 165

length of stay 931

letrozole 1815

leukaemia 879

life expectancy 778

lifestyle 201

lipoprotein lipase 739

liver cancer 195, 334

liver perfusion 658

living alone 189

LMP2 53

LNCaP 1714

loss of heterozygosity 1776

low grade 575

low-income countries 183

luminal membrane expression 137

lung adenocarcinoma 1745

lung cancer 442, 1083, 1188, 1361, 1534, 1584, 1820

lymphadenectomy 785

lymphangiogenesis 700

lymphatic metastasis 150

lymphocytes 695, 864

lymphoma 879

M30 1286

macrophage migration inhibitory factor 1498

magnetic resonance imaging 24

malignant glioma 1481

malignant PE 1876

malignant tumours 1761

mammographic density 18

mammography 24

mass screening/methods 1051

mathematical modelling 814

MCM-2 1384

mechanistic PK/PD 814

*MED12* 1761

medulloblastoma 1144, 1399

melanoma 422, 455, 570, 977, 1498

men 201

meningioma 1702

menopausal hormone therapy 1181

mesothelin 137

mesothelioma 161, 1107, 1978

MET 325

*MET* 793

meta-analysis 1163, 1608

metabolism 1207

metachronous cancer 221

metaplasia–adenoma–carcinoma sequence 1125

metastasectomy 422

metastasis 63, 75, 129, 360, 375, 707, 724, 732, 977, 1059, 1302, 1547, 1678, 1737, 1944, 1963

metastasised colorectal cancer 961

metastatic breast cancer 1454

metastatic colorectal cancer 1932, 1950

methotrexate resistance 1810

methylation 100, 732, 1423

microarray 91, 684, 1361

microRNA 123, 352, 684, 1345, 1754, 1987

microvessel density 334

middle-income countries 183

MIF 1498, 1595

migration 1317, 847

miR-126 700

miR-204 967

miRNA 1361

miRNA-21 1169

miRNAs 1354

mismatch repair 1399

mismatch repair genes 1783

mitochondria DNA 2016

mitotic inhibitor 1692

mixed lineage leukaemia 315

MLH1 1783

MLL4 315

MLL-antisense 315

MMP-9 1554

MMPs 143

molar pregnancy 1810

molecular biomarkers 1069

molecular diagnostics 345, 1138

molecular subtypes 18, 382

mortality 1901

mother 544

MSH2 1783

MSH6 1783

mTOR inhibitors 1093

multiple myeloma 1844

multiplex bead assays 287

mutation 1997

mutation screening 1761

Myc 1144

myelodysplastic/myeloproliferative neoplasms 879

myeloma 1987

NAC1 300

*N*-acetylglucosaminyltransferase-IV 1969

nasopharyngeal carcinoma 1580

nass 1618

National Cancer Institute 1950

national registries 176

NCAD 1374

NEAT 1257

neoadjuvant 7

neoadjuvant chemoradiotherapy 1925

neoadjuvant chemotherapy 1233, 1892, 1925

neoadjuvant therapy 956, 961

neoplasm staging 150

neuroblastoma 967, 1409, 1418

neuronal differentiation 1144

neutropenia 814

neutrophil 695

NF*κ*B 1488, 1554

NGR-hTNF 37

nomogram 918, 1031, 1467, 1826

noncancerous tissues 1963

non-Hodgkin lymphoma 1423

non-sentinel lymph node 1239

non-small cell lung cancer 823, 1107, 1277, 1361, 1474

non-steroidal anti-inflammatory drugs 207

normal tissue radiation injury 748

North America 1917

Notch 1207, 1374

novel sequence 388

NSAID 1602

NT5E 75

NTRK2 967

nuclear factor-*κ*B 652

nuclear receptor 831

nutrients 1580

obesity 169

oesophageal adenocarcinoma 564, 1766

oesophageal cancer 143, 1584, 1618, 1908, 1925

oesophageal neoplasm 888

oesophagectomy 1925

oesophago-gastric cancer 435

oestrogen receptor *β* 831

oestrogen receptor status 165

omega-6 1737

oncological trial 1

oncology 695, 1022, 1801

one-step nucleic amplification assay 1239

optimisation 814

oral hygiene 888

oral squamous cell carcinoma 700

orlistat 977

osteonecrosis of the jaw 1665

osteopontin 840, 1131

outcome 1392, 1665

ovarian cancer 37, 360, 785, 925, 1069, 1181, 1327, 1776, 1869

ovarian clear cell carcinoma 300

ovarian serous adenocarcinoma 91

overall survival 266, 588, 1454

oxaliplatin 340, 1950

OxCap 1037

OxFU 1037

p16 cellular localisation 482

p53 334, 516

paclitaxel 31, 1268, 360, 588, 823, 1692

Paget's disease 646

palliative chemotherapy 442

PAM 1409

pancreatic cancer 280, 411, 501, 632, 1354, 1692, 2005

pancreatic head cancer 1153

pancreatic neoplasms 1883

pancreaticoduodenectomy 1883

paracentesis 925

parity 564

paternal occupation 1652

paternity 1833

pathology 1467

pathways 1220

patterns of metastasis 221

Pax8 370

pazopanib 639

PCR 116, 1392

PDGFR*β* 1702

pegylated liposomal doxorubicin 588

pemetrexed 604

pepsinogen 888

performance 1025

periampullary cancer 1153

peroxisome biogenesis factor-5 739

persistence 1249

persistent pain 1459

personalised therapy 1820

PGE_2_ 1514

PGT 707, 1514

pharmacokinetics 429, 455, 604, 1277, 1525

phase I 604, 1022, 1093

phase I oncology trials 1025

phase I study 429, 612, 1474

phase II 280, 808

phase II design 1801

phase II study 1474

pHH3 84

photoreaction 1534

pI*κ*B*α* 1554

PIM kinases 491

plasma cell neoplasms 879

plasma cells 864

platelet lymphocyte ratio 1525

platelets 1564

platinum 1637

platinum drugs 1327

platinum-sensitive 588

PleureX 925

PLK1 1766

Plk1 inhibitor 280

polyADP-ribosylation 1317

polymorphism 1840

population mixing 1163

population study 1169

population-based 12, 161, 570

postmenopausal 1815

postoperative pain 937

pre-clinical cancer 527

precore mutant 2010

prediction 1009, 1409, 1826

prediction model 918

predictive biomarkers 675, 1233, 1547

predictive factor 1892

prednisone 808

preeclampsia 176

pregnancy 176

preinvasive lesions 1451

prenatal hormones 1631

prenatal stress 544

prevention and control 1051

primary care 1213, 1644

primary central nervous system lymphoma 1840

primary ductal invasive breast cancer 864

*PRKCZ* 388

PRL-3 352

profiling 684, 1433

prognosis 84, 100, 150, 108, 116, 334, 422, 508, 639, 695, 831, 918, 1107, 1131, 1138, 1153, 1418, 1433, 1454, 1518, 1637, 1672, 1883, 1997

prognostic biomarker 370, 667

prognostic factors 161, 632, 1009, 1227

prognostic marker 1169

prognostic model 800

prognostic score 275

progression 1059, 1392

projections 1195

proliferation 508

Prolyl hydroxylases 1423

propionate 1337

prospective cohort 831, 895

prostate cancer 100, 207, 388, 575, 592, 646, 652, 713, 778, 840, 847, 895, 1467, 1547, 1564, 1584, 1714, 1737, 1963

prostatectomy 1963

prostatic neoplasms 808

proteomics 1820

PSA 100

psychological factors 937

pyridoxine 585

pyropheophorbide-a 1534

QCancer 260

qRT–PCR 1354

quality of life 904, 1295

quantitative RT–PCR 150

RAD001 resistance 847

radiation 291, 308, 652, 994

radical cystectomy 1826

radical prostatectomy 1467

radiosensitizers 469

radiotherapy 531, 549, 840, 1361

randomised trial 585, 1917

RB 516

RCC 1131

reactive oxygen species 1488

rectal cancer 266

recurrence 1138

recurrent ovarian cancer 588

reduced tumour burden 501

referral 1220

rehabilitation care 931

relative risk 888

renal cancer 856, 1131, 1227, 1286

renal cell carcinoma 1009, 1059, 1665

renal function 1037

replication 291

residual cancer 1138

resistance 1853

reverse causation 527

rhabdomyosarcoma 1374

risk 549

risk factors 1459

risk prediction 260

route 1220

RPN2 1233

rs2736100 1001

RUNX2 1714

S-1 1474

S-1 31

S100 422

S100A4 667

sample size 1801

sarcopenia 931

Scotland 575

screening 24, 417, 757, 765, 778, 1917

screening for distress 617

screening tool 1459

screen-specific anxiety 1295

second malignancies 531

second-look surgery 785

secreted SEMA5A 501

semaphorin 501

seminoma 1754

sensitivity and specificity 24

sentinel lymph node 724

serum 956, 1729

serum biomarkers 287, 1820

serum miRNAs 1987

sex hormone 564

sex steroids 831

shear wave 224

short term survival 1244

side effects 158

signalling 1317

silica 1188

silver *in-situ* hybridisation 325

simulation 1801

single-visit approach 772

sitting height 165

skin 1678

skin rash 1797

sleep disturbance 527

SMAD signalling 1978

small cell lung cancer 1361

smoking 234, 537, 879, 1618

snoRNA 684

social class 1652

social support 189

socio-economic deprivation 1213

socioeconomic factors 12

socio-economic inequalities 575

socioeconomic status 189, 765

soft-tissue sarcomas 639, 1374

solid tumours 1268, 1277

somatic mutation 1761

sorafenib 455, 592, 1820

spermatogenesis 1833

Src 856

Src kinase 856

stage II 1169

staging 12, 1672

standard chemotherapy 108

STAT3 352

statistical analysis 508

stomach neoplasm 325, 537

structural variant 388

sub-Saharan Africa 556

subtypes 895

sufficient life expectancy 1025

sunitinib 1286

surgery 840, 918, 931, 1175

survival 91, 161, 275, 300, 334, 537, 757, 864, 874, 918, 1672, 1722, 1840, 1883, 1059, 1075, 1220, 1327, 2005

survival analysis 531, 695

survival prediction 1031

survivin 84

survivors 1195

survivorship 1195

symptoms 904

systematic review 243

T cells 1107, 1869

T1-3 N0 1826

tamoxifen 230

tamoxifen resistance 43, 947

tandutinib 1702

target population 1445

target validation 53

targeted therapy 947, 1227, 1665, 1978

TAS-102 429

tea 874

telomerase 448, 1844

telomerase regulation 1844

telomere 1844

telomere length 1525

temozolomide 1399

*TERT–CLPTM1L* 1001

testicular cancer 1833

testicular germ cell tumour 1754, 1853

TFT 429

TGF-*β*-activated kinase-1 129

the Glasgow Prognostic Score 988

The Netherlands 12

therapeutic target 63

third primary cancer 549

thymosin beta 15 1892

thyroid 994

TIGAR 516

time trends 1159

tissue damage 1534

tissue factor 1125

tissue microarray 482

tobacco 1618

topoisomerase 2 108

toxic death 1

toxicity 158, 308

*TP53* mutation 1138

*TP53* mutation status 1722

TPI 429

traditional Chinese medicine 411

trastuzumab 956, 1075

TRC 724

treatment 12, 1901

treatment response 1684

triage 617

triple-negative breast cancer 75, 1892

trust 1938

tumour angiogenesis 1853

tumour burden 1518

tumour burden and metastasis 501

tumour immunology 1729

tumour inflammatory cell infiltrate 864

tumour margin 462

tumour necrosis factor alpha (*TNFα*). 748

tumour regression 516

tumour suppression 315

tumour suppressor 967

tumour xenograft 315

tumour-initiating cells 462

tyrosine kinase inhibitors 1227

ultrasound 224, 469

United Kingdom 1195, 1467

uranium miners 1188

urinary bladder neoplasms 1589

urine 123

urothelial carcinoma 1384

US Veterans Affairs 195

utility 778

validation 260

vascular endothelial growth factor signalling 1722

vascular targeting agent 37

vatalanib 1044

VE-821 291

VEGF 961, 1207, 1481, 1702, 1869

VEGF trap 598

VEGF-A 700, 977

VEGFR2 1044

VEGFR-2 1869

venous thromboembolism 612

vinorelbine 442, 823

visual inspection with acetic acid (VIA) 772

vitamin D 158

vitamin D binding protein 1589

whole sentinel lymph node 1239

WNT pathway 1144

wound healing 961

young age 382

zoledronic acid 7

